# Supplementary material for: Gears in chemical reaction networks for optimizing energy transduction efficiency
Source: Nat Commun. 2025 Jul 1;16:5765. doi: 10.1038/s41467-025-60787-1 (PMC12215990; doi:10.1038/s41467-025-60787-1)
Supplement: Supplementary file 1 — Supplementary Information [file 41467_2025_60787_MOESM1_ESM.pdf]

Supplementary Information for "Gears in Chemical Reaction Networks: Optimizing Energy Transduction Efficiency"

Massimo Bilancioni[1] and Massimiliano Esposito[1]

[1] *Complex Systems and Statistical Mechanics, Department of Physics and Materials Science, University of Luxembourg, 30 Avenue des Hauts-Fourneaux, L-4362 Esch-sur-Alzette, Luxembourg*

Contents

I. CRN cycles 1
A. Internal, external, and emergent cycles 2
B. Steady state flux and entropy production 2

II. CRN Processes 3

III. Elementary flux modes 3

IV. CRN Gears 3
A. Relation between external EFMs and emergent cycles 4

V. Steady state flux and entropy production in terms of conformal gears 4
A. Chemical current 4
B. Entropy production 5

VI. Proof of the upper bound on the transduction efficiency 6

VII. Self-regulating CRN 7

References 8

I. CRN CYCLES

We considered the setup of the paper: an open CRN with internal species X, external species Y and a set of reactions ρ. The concentrations of the X and Y species are respectively denoted by $\boldsymbol{x}$ and $\boldsymbol{y}$ and the stoichiometric matrices reduced to the X and Y species are respectively denoted by $\mathbb{S}^X$ and $\mathbb{S}^Y$. For the CRN in Fig. S1a, we have:

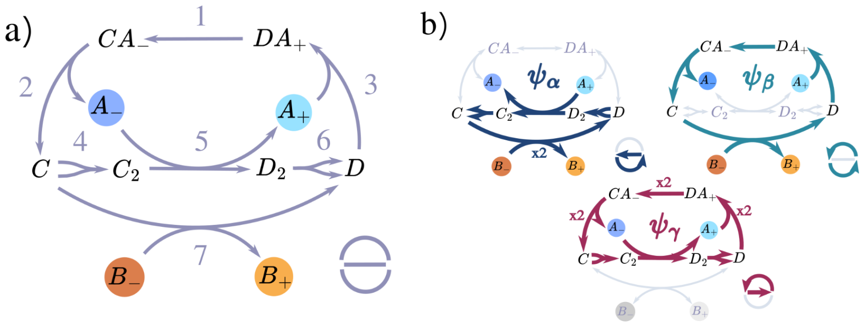

FIG. S1: **a)** Illustrative CRN reported from the main text: we detail the enumeration and forward direction of reactions. **b)** The three gear cycles of the CRN.

$$\mathbb{S} = \left(\begin{array}{c|ccccccc} & 1 & 2 & 3 & 4 & 5 & 6 & 7 \\ \hline A_- & 0 & +1 & 0 & 0 & -1 & 0 & 0 \\ A_+ & 0 & 0 & -1 & 0 & +1 & 0 & 0 \\ B_- & 0 & 0 & 0 & 0 & 0 & 0 & -1 \\ B_+ & 0 & 0 & 0 & 0 & 0 & 0 & +1 \\ CA_- & +1 & -1 & 0 & 0 & 0 & 0 & 0 \\ C & 0 & +1 & 0 & -2 & 0 & 0 & -1 \\ C_2 & 0 & 0 & 0 & +1 & -1 & 0 & 0 \\ DA_+ & -1 & 0 & +1 & 0 & 0 & 0 & 0 \\ D & 0 & 0 & -1 & 0 & 0 & +2 & +1 \\ D_2 & 0 & 0 & 0 & 0 & +1 & -1 & 0 \end{array}\right) \begin{array}{l} \left.\vphantom{\begin{array}{c}0\\0\\0\\0\end{array}}\right\} \mathbb{S}^Y \\ \left.\vphantom{\begin{array}{c}0\\0\\0\\0\\0\\0\end{array}}\right\} \mathbb{S}^X \end{array} . \tag{S1}$$

These matrices specify the dynamics of the X and Y species: $\dot{\boldsymbol{x}} = \mathbb{S}^X \boldsymbol{J}$ and $\dot{\boldsymbol{y}} = \mathbb{S}^Y \boldsymbol{J} + \boldsymbol{I}^Y$, where $\boldsymbol{J}$ is the vector of the reaction fluxes and $\boldsymbol{I}^Y$ is the flux with which the Y species enter the CRN from the surrounding. Similarly, if $\boldsymbol{v}$ is a vector in the space of reactions whose entries v_ρ denote the number of times each reaction ρ occurs, the same matrices describe how $\boldsymbol{v}$ affects the species' concentrations:

$$\Delta \boldsymbol{x} = \mathbb{S}^X \boldsymbol{v}, \qquad \Delta \boldsymbol{y} = \mathbb{S}^Y \boldsymbol{v}. \tag{S2}$$

A CRN *cycle* is defined as a vector $\boldsymbol{\psi}$ that leaves the concentration of the internal species unchanged.

$$\mathbb{S}^X \boldsymbol{\psi} = 0. \tag{S3}$$

For the previous CRN, $\boldsymbol{\psi}_\alpha$, $\boldsymbol{\psi}_\beta$, $\boldsymbol{\psi}_\gamma$ in Fig. S1b, as well as any linear combinations of these vectors, are cycles.

$$\boldsymbol{\psi}_\alpha = \begin{array}{c}1\\2\\3\\4\\5\\6\\7\end{array} \begin{pmatrix} 0 \\ 0 \\ 0 \\ -1 \\ -1 \\ -1 \\ +2 \end{pmatrix}, \quad \boldsymbol{\psi}_\beta = \begin{array}{c}1\\2\\3\\4\\5\\6\\7\end{array} \begin{pmatrix} +1 \\ +1 \\ +1 \\ 0 \\ 0 \\ 0 \\ +1 \end{pmatrix}, \quad \boldsymbol{\psi}_\gamma = \begin{array}{c}1\\2\\3\\4\\5\\6\\7\end{array} \begin{pmatrix} +2 \\ +2 \\ +2 \\ +1 \\ +1 \\ +1 \\ 0 \end{pmatrix}. \tag{S4}$$

A. Internal, external, and emergent cycles

An *internal cycle* is a cycle that has no net effect on the Y species, $\mathbb{S}^Y \boldsymbol{\phi} = 0$, while an *external cycle* acts as an effective reaction on the Y species: $\mathbb{S}^Y \boldsymbol{\phi} \neq 0$.

Let us define a set of independent internal cycles $\{\boldsymbol{\phi}_i\}$. By definition, they satisfy $\mathbb{S}\, \boldsymbol{\phi}_i = 0$ and their number is thus $N_i = \dim(\ker(\mathbb{S}))$. We also define an additional set of independent vectors $\{\boldsymbol{\phi}_\epsilon\}$ that, together with $\{\boldsymbol{\phi}_i\}$, form a basis for the cycles of $\mathbb{S}^X$. We call them *emergent cycles* [1] and their number is $N_\epsilon = \dim(\ker(\mathbb{S}^X)) - \dim(\ker(\mathbb{S}))$. Emergent cycles are by construction external cycles as $\mathbb{S}^Y \boldsymbol{\phi}_\epsilon \neq 0$. The choice of both internal and emergent cycles is arbitrary. The CRN in Fig. S1a has only two emergent cycles that can be freely chosen as any pair among $\boldsymbol{\psi}_\alpha$, $\boldsymbol{\psi}_\beta$, $\boldsymbol{\psi}_\gamma$, or two independent linear combinations of these.

B. Steady state flux and entropy production

From now on, we consider open CRNs in stationary conditions and all quantities are implicitly interpreted as steady state quantities. The steady state fluxes $\boldsymbol{J}$ of the open CRN, defined by $0 = \dot{\boldsymbol{x}} = \mathbb{S}^X \boldsymbol{J}$, are cycles which can be decomposed any CRN steady state flux $\boldsymbol{J}$:

$$\boldsymbol{J} = \sum_i J_i \boldsymbol{\phi}_i + \sum_\epsilon J_\epsilon \boldsymbol{\phi}_\epsilon. \tag{S5}$$

As a result, the steady state entropy production can be expressed solely in terms of emergent cycles

$$\dot{\Sigma} = -\boldsymbol{J} \cdot \Delta \boldsymbol{G} = -\sum_i \cancel{J_i \boldsymbol{\phi}_i \cdot \Delta \boldsymbol{G}} - \sum_\epsilon J_\epsilon \boldsymbol{\phi}_\epsilon \cdot \Delta \boldsymbol{G} = -\sum_\epsilon J_\epsilon \Delta_\epsilon G \geq 0. \tag{S6}$$

Here, $\Delta \boldsymbol{G} = \boldsymbol{\mu}\,\mathbb{S}$ is the vector that encodes the Gibbs free energy change of each reaction ρ defined in terms of $\boldsymbol{\mu}$, the vector of chemical potentials. Therefore, $\Delta_\epsilon G = \Delta \boldsymbol{G} \cdot \boldsymbol{\phi}_\epsilon$ is the Gibbs free energy change associated to the emergent cycle ϵ which only depends on the chemical potentials of the Y species, since $\Delta_\epsilon G = \boldsymbol{\mu}\,\mathbb{S}\,\boldsymbol{\phi}_\epsilon = \boldsymbol{\mu}_Y \mathbb{S}^Y \boldsymbol{\phi}_\epsilon$. Instead, the Gibbs free energy changes associated to internal cycles are zero because internal cycles do not affect any species concentration. For the CRN of Fig. S1, if we choose $\{\boldsymbol{\psi}_\beta,\, \boldsymbol{\psi}_\gamma\}$ as emergent cycles, one can write:

$$\boldsymbol{J} = J_\beta \boldsymbol{\psi}_\beta + J_\gamma \boldsymbol{\psi}_\gamma \quad \text{and} \quad \dot{\Sigma} = -J_\beta \Delta_\beta G - J_\gamma \Delta_\gamma G, \tag{S7}$$

with $\Delta_\beta G = (\mu_{A_-} - \mu_{A_+}) + (\mu_{B_+} - \mu_{B_-})$ and $\Delta_\gamma G = (\mu_{A_-} - \mu_{A_+})$.

II. CRN PROCESSES

A *chemical process* is an effective reaction among the Y species that is stochiometrically balanced with respect to charge and atomic nuclei. In the paper, we make the following assumption concerning chemical processes: the net reaction arising from any cycle $\boldsymbol{\psi}$ can be decomposed in terms of only two processes $\boldsymbol{p}_a$ and $\boldsymbol{p}_b$. Mathematically, for any $\boldsymbol{\psi}$, there are m_a^ψ and m_b^ψ such that

$$\boldsymbol{p}_\psi = \mathbb{S}^Y \boldsymbol{\psi} = m_a^\psi\, \boldsymbol{p}_a + m_b^\psi\, \boldsymbol{p}_b. \tag{S8}$$

At steady state, given that $\dot{\boldsymbol{y}} = 0$ and $\boldsymbol{J}$ is a cycle, the flux of Y entering the CRN can be decomposed as:

$$\boldsymbol{I}^Y = -\mathbb{S}^Y \boldsymbol{J} = -\mathcal{I}_a\, \boldsymbol{p}_a - \mathcal{I}_b\, \boldsymbol{p}_b, \tag{S9}$$

where $\mathcal{I}_a$ and $\mathcal{I}_b$ represent the rates at which process a and b are produced in the system. As a result, the entropy production in the environment can be expressed as

$$\dot{\Sigma} = \boldsymbol{\mu}_Y \cdot \boldsymbol{I}^Y = -\mathcal{I}_a\, \boldsymbol{\mu}_Y \cdot \boldsymbol{p}_a - \mathcal{I}_b\, \boldsymbol{\mu}_Y \cdot \boldsymbol{p}_b = -\mathcal{I}_a\, \Delta_a G - \mathcal{I}_b\, \Delta_b G > 0, \tag{S10}$$

where $\Delta_a G = \boldsymbol{\mu}_Y \cdot \boldsymbol{p}_a$ and $\Delta_b G = \boldsymbol{\mu}_Y \cdot \boldsymbol{p}_b$ are the Gibbs free energy changes associated to the two processes. For the CRN in Fig. S1,

$$\boldsymbol{p}_a = \begin{matrix} A_- \\ A_+ \\ B_- \\ B_+ \end{matrix} \begin{pmatrix} +1 \\ -1 \\ 0 \\ 0 \end{pmatrix} \quad \text{and} \quad \boldsymbol{p}_b = \begin{matrix} A_- \\ A_+ \\ B_- \\ B_+ \end{matrix} \begin{pmatrix} 0 \\ 0 \\ +1 \\ -1 \end{pmatrix}, \tag{S11}$$

with the associated Gibbs free energy changes $\Delta_a G = \mu_{A_-} - \mu_{A_+}$ and $\Delta_b G = \mu_{B_-} - \mu_{B_+}$.

III. ELEMENTARY FLUX MODES

An *elementary flux mode* (EFM) is a special kind of cycle that uses a minimal *set* of reactions. More precisely, a cycle $\boldsymbol{\psi}$ is an EFM if there is no other cycle $\boldsymbol{\psi}'$ such that $\mathrm{supp}(\boldsymbol{\psi}') \subset \mathrm{supp}(\boldsymbol{\psi})$, where $\mathrm{supp}(\boldsymbol{v})$ denotes the support of a vector that is equal to the set of reactions for which $v_\rho \neq 0$. A direct consequence of support minimality is that, if $\boldsymbol{\psi}$ is an EFM, $\mathrm{supp}(\boldsymbol{\psi})$ defines a unicyclic subnetwork, a network that has only one independent cycle. Indeed, if that were not the case, one could remove one reaction and create with the remaining subset a smaller cycle, thus violating this condition. As for cycles, they can be *internal* EFMs if they do not affect the Y species or *external* EFMs if they create an effective reaction on the Y species. The cycles $\boldsymbol{\psi}_\alpha$, $\boldsymbol{\psi}_\beta$, and $\boldsymbol{\psi}_\gamma$, in Eq. (S4), constitute the full set of EFMs of the CRN in Fig. S1.

Algorithms have been developed to enumerate EFMs [2], but the complete enumeration of EFMs becomes computationally intensive for large metabolic CRNs [3], as their number grows exponentially with the size of the network.

IV. CRN GEARS

The *gears* $\boldsymbol{\psi}_g$ of an open CRN are its external EFMs. As a result of the assumption in Eq. (S8), the effect of a gear on the Y concentrations can be expressed in terms of two processes $\boldsymbol{p}_a$ and $\boldsymbol{p}_b$:

$$\mathbb{S}^Y \boldsymbol{\psi}_g = m_a^{\mathrm{g}}\, \boldsymbol{p}_a + m_b^{\mathrm{g}}\, \boldsymbol{p}_b. \tag{S12}$$

The Gibbs free energy change associated to each gear $\boldsymbol{\psi}_g$ is then

$$\Delta_g G = \boldsymbol{\mu}_Y \cdot \mathbb{S}^Y \boldsymbol{\psi}_g = m_a^{\mathfrak{g}} \boldsymbol{\mu}_Y \cdot \boldsymbol{p}_a + m_b^{\mathfrak{g}} \boldsymbol{\mu}_Y \cdot \boldsymbol{p}_b = m_a^{\mathfrak{g}} \Delta_a G + m_b^{\mathfrak{g}} \Delta_b G, \tag{S13}$$

and the ratio between the last two terms gives the efficiency of the gear defined in Eq. (3). The CRN in Fig. S1 has three gears: the external EFMs $\boldsymbol{\psi}_\alpha$, $\boldsymbol{\psi}_\beta$ and $\boldsymbol{\psi}_\gamma$; and their effect on the Y species can be written as:

$$\begin{aligned} \mathbb{S}^Y \boldsymbol{\psi}_\alpha &= 2\boldsymbol{p}_a - \boldsymbol{p}_b, \\ \mathbb{S}^Y \boldsymbol{\psi}_\beta &= \boldsymbol{p}_a - \boldsymbol{p}_b, \\ \mathbb{S}^Y \boldsymbol{\psi}_\gamma &= \boldsymbol{p}_a. \end{aligned} \tag{S14}$$

Since gears are external EFMs, it may be possible to design faster algorithms that target them directly, avoiding internal EFMs.

A. Relation between external EFMs and emergent cycles

Internal EFMs are vectors lying in ker($\mathbb{S}$) and therefore linear combinations of the internal cycles $\{\boldsymbol{\phi}_i\}$. The number of independent internal EFMs is $\leq$ than the number of internal cycles, equal to N_i= dim(ker($\mathbb{S}$)) (see Sect. I). External EFMs lie instead in ker ($\mathbb{S}^X$) and are generally linear combinations of both internal and emergent cycles. Since all EFMs together span ker($\mathbb{S}^X$), the number of independent external EFMs is $\geq$ than the number of emergent cycles N_ϵ = dim(ker($\mathbb{S}^X$)) $-$ dim(ker($\mathbb{S}$)) (see Sect. I).

V. STEADY STATE FLUX AND ENTROPY PRODUCTION IN TERMS OF CONFORMAL GEARS

In this section, we first revisit a known mathematical result [4] that we then exploit to derive a thermodynamically meaningful decomposition for the entropy production. This decomposition will allow us to prove the upper bound on the transduction efficiency in Sect. VI. We start by introducing some essential mathematical definitions.

Definition 1. *A vector $\boldsymbol{\varphi}$ is conformal to $\boldsymbol{\phi}$ if $\varphi_\rho \neq 0 \implies sgn(\varphi_\rho) = sgn(\phi_\rho)$ for any component ρ.*

Geometrically, $\boldsymbol{\varphi}$ is conformal to $\boldsymbol{\phi}$ if it belongs to the same hyperoctant. Note that, due to zero components, this relation may not be reciprocal: $\boldsymbol{\varphi}$ conf to $\boldsymbol{\phi}$ $\not\Longrightarrow$ $\boldsymbol{\phi}$ conf to $\boldsymbol{\varphi}$.

Definition 2. *$\boldsymbol{\phi} = \sum_k \boldsymbol{\varphi}_k$ is a conformal decomposition if all vectors $\boldsymbol{\varphi}_k$ are conformal to $\boldsymbol{\phi}$.*

One can think of it as a decomposition without cancelation: for any component ρ (i.e., a specific coordinate or entry in the vector representation), each term $\varphi_{k\rho}$ (the ρ-th entry of vector $\boldsymbol{\varphi}_k$) is either zero or shares the same sign as ϕ_ρ.

A. Chemical current

The main result is that any steady state current $\boldsymbol{J}$ flowing in the CRN can be decomposed in terms of conformal EFMs, i.e.

$$\boldsymbol{J} = \sum_c j_c \boldsymbol{\psi}_c, \tag{S15}$$

where the vectors $j_c\boldsymbol{\psi}_c$ are conformal to $\boldsymbol{J}$ and linearly independent; see the proof below. This independence guarantees that the number of gears required to decompose any current is $\leq$ dim(ker($\mathbb{S}^X$)). However, we stress that the set of gears used varies with the specific $\boldsymbol{J}$ and that this decomposition may not be unique. When applied to the CRN of Fig. S1a, we find that any stationary current can be conformally decomposed using at most two gears; see Fig. (S2) for an example.

The proof is readapted from [4] and proceeds by induction on the cardinality of supp($\boldsymbol{J}$), making use of the following lemma.

Lemma 1. *Given a cycle $\boldsymbol{\psi} \in ker(\mathbb{S}^X)$, one can always find a EFM conformal to it.*

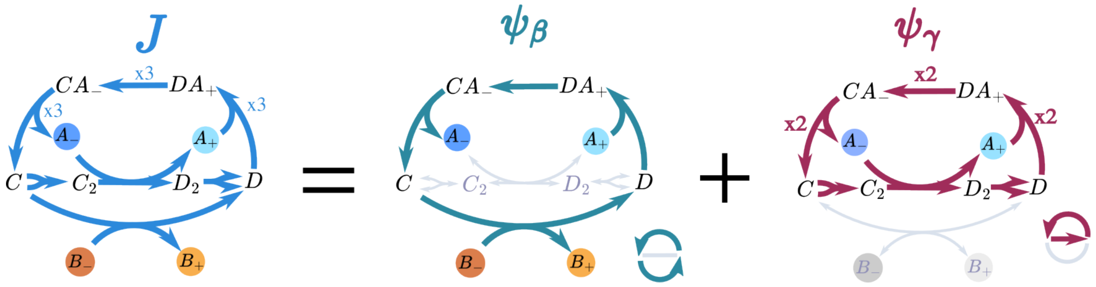

FIG. S2: Decomposition of the chemical current $\boldsymbol{J}$ of CRN in Fig. 1b in terms of the conformal EFMs $\boldsymbol{\psi}_\beta$ and $\boldsymbol{\psi}_\gamma$.

Proof (Lemma). If $\boldsymbol{\psi}$ is an EFM, the lemma is trivially true. Otherwise, we show that one can build a vector $\boldsymbol{\psi}'' \in \ker(\mathbb{S}^X)$ conformal to $\boldsymbol{\psi}$ that has a smaller support. When $\boldsymbol{\psi}$ is not an EFM, it is, by definition, not support minimal. Thus, there exists $\boldsymbol{\psi}' \in \ker(\mathbb{S}^X)$ such that $\mathrm{supp}(\boldsymbol{\psi}') \subset \mathrm{supp}(\boldsymbol{\psi})$. If $\pm\boldsymbol{\psi}'$ is conformal to $\boldsymbol{\psi}$, then $\boldsymbol{\psi}'' = \pm\boldsymbol{\psi}'$. If not, $\boldsymbol{\psi}'' = \boldsymbol{\psi} - \lambda\boldsymbol{\psi}'$ where $\lambda > 0$ is the highest value for which $\boldsymbol{\psi}''$ is still conformal to $\boldsymbol{\psi}$. By subtracting $\lambda\boldsymbol{\psi}'$, we cancel a component of $\boldsymbol{\psi}$ resulting in $\mathrm{supp}(\boldsymbol{\psi}'') \subset \mathrm{supp}(\boldsymbol{\psi})$. Now, if $\boldsymbol{\psi}''$ is a conformal EFM, the lemma is satisfied; otherwise, we can repeat the above procedure until the final vector is support minimal and thus a conformal EFM. □

Proof (Current's conformal decomposition). Since $\boldsymbol{J} \in \ker(\mathbb{S}^X)$, the lemma ensures the existence of a conformal EFM $\boldsymbol{\psi}_c$. Through $\boldsymbol{\psi}_c$, we can build a vector $\boldsymbol{J}^*$ conformal to $\boldsymbol{J}$ such that $\mathrm{supp}(\boldsymbol{J}^*) \subset \mathrm{supp}(\boldsymbol{J})$. It suffices to choose, in the expression $\boldsymbol{J}^* = \boldsymbol{J} - j_c^*\boldsymbol{\psi}_c$, the maximum $j_c^* > 0$ for which $\boldsymbol{J}^*$ is still conformal to $\boldsymbol{J}$. Then, the induction hypothesis tells us that $\boldsymbol{J}^*$ admits a conformal decomposition in terms of linearly independent conformal EFMs: $\boldsymbol{J}^* = \sum_{c'} j_{c'}\boldsymbol{\psi}_{c'}$. Since $\mathrm{supp}(\boldsymbol{\psi}_c) \not\subset \mathrm{supp}(\boldsymbol{J}^*)$, $\boldsymbol{\psi}_c$ is also independent of all the other $\boldsymbol{\psi}_{c'}$. Therefore, the desired conformal decomposition for $\boldsymbol{J}$ follows:

$$\boldsymbol{J} = j_c^*\boldsymbol{\psi}_c + \boldsymbol{J}^* = j_c^*\boldsymbol{\psi}_c + \sum_{c'} j_{c'}\boldsymbol{\psi}_{c'}. \tag{S16}$$

□

B. Entropy production

Using Eq. (S15), we can now decompose the stationary entropy production in terms of conformal gears (external EFMs). We assume the *local* validity of the second law, i.e. $\dot{\Sigma}_\rho = -J_\rho\Delta_\rho G \geq 0$ and $-\Delta_\rho G = 0 \iff J_\rho = 0$ for any ρ (with finite reaction rates). $-\Delta G_\rho$ is the Gibbs free energy change of reaction ρ. In mathematical terms, this assumption is equivalent to saying that $\boldsymbol{J}$ and $-\boldsymbol{\Delta G}$ are reciprocally conformal. Using Eq. (S15), the entropy production can be decomposed as

$$\dot{\Sigma} = -\boldsymbol{J}\cdot\boldsymbol{\Delta G} = -\sum_c j_c\boldsymbol{\psi}_c\cdot\boldsymbol{\Delta G}. \tag{S17}$$

By transitivity, each $j_c\boldsymbol{\psi}_c$, in addition to being conformal to $\boldsymbol{J}$, is also conformal to $-\boldsymbol{\Delta G}$. Therefore, if we denote by $\Delta_c G = \boldsymbol{\psi}_c\cdot\boldsymbol{\Delta G}$ the Gibbs free energy change associated to the conformal EFMs, it is:

$$\dot{\Sigma} = \sum_c \dot{\Sigma}_c = -\sum_c j_c\Delta_c G \quad \text{with} \quad \dot{\Sigma}_c = -j_c\Delta_c G > 0 \quad \text{for every } c. \tag{S18}$$

From this decomposition, we see that the conformal EFMs in Eq. (S15) are also gears since they need to be external: $\Delta_c G \neq 0$ is possible only if the EFMs alter the concentrations of the Y species (see Sect. IV). It is interesting to compare Eq. (S18) with the alternative entropy production decompositions in terms of reactions, $\dot{\Sigma} = -\sum_\rho J_\rho\Delta_\rho G$, and in terms of emergent cycles, Eq. (S6). When decomposed in terms of reactions, all terms $-J_\rho\Delta_\rho G$ are individually nonnegative, but the Gibbs free energy changes $\Delta_\rho G$ can depend on the chemical potentials μ_X of the internal species: For example, reaction 7 in Fig. S1a has $\Delta_7 G = \mu_D - \mu_C + \mu_{B_+} - \mu_{B_-}$. Instead, the Gibbs free energy changes $\Delta_\epsilon G$ of emergent cycles depend only on μ_Y, but the individual terms in Eq. (S6) are no longer guaranteed to be nonnegative. Our new decomposition, Eq. (S18), combines the advantages of the previous two: it ensures $-j_c\Delta_c G > 0$ for every conformal gear and $\Delta_c G = \boldsymbol{\mu}_Y\mathbb{S}^Y\boldsymbol{\psi}_c$ depends only on the external μ_Y. However, we note that the set of emergent

cycles in Eq. (S6) has dimension $N_\epsilon = \dim(\ker(\mathbb{S}^X)) - \dim(\ker(\mathbb{S}))$ (see Sect. IV A) and can be fixed once and for all. Instead, in Eq. (S18), the set of conformal gears is at most of dimension $\dim(\ker(\mathbb{S}^X))$ and varies with the specific $\boldsymbol{J}$, which changes, for example, when the values of the chemical potentials μ_Y change. Finally, we note that when the CRN reduced to the X species defines a linear network, Hill found in Ref. [5] an entropy production decomposition similar to Eq. (S18), but where *all* gears appear. In that case, the gears correspond to the graph closed paths, and their coefficients to the net rates at which these closed paths are run under stationary conditions.

VI. PROOF OF THE UPPER BOUND ON THE TRANSDUCTION EFFICIENCY

We prove that

$$\eta \leq \eta_{\max}\left(\frac{\Delta_b G}{\Delta_a G}\right) = \max_{\eta_g < 1} \eta_g. \tag{S19}$$

First of all, we rewrite the efficiency

$$\eta = \frac{\mathcal{I}_b \Delta_b G}{-\mathcal{I}_a \Delta_a G} \tag{S20}$$

in terms of the efficiencies of the conformal gears η_c and the associated currents j_c. To this aim, we note that we can write the stationary rates at which the two processes occur as

$$\mathcal{I}_{a/b} = \sum_c j_c m^c_{a/b}, \tag{S21}$$

where we recall that $m^c_{a/b}$ are the one given by Eq. (S8) applied to gear c. Substituting Eq. (S21) into η, one obtains

$$\eta = \frac{\sum_c j_c m^c_b \Delta_b G}{-\sum_c j_c m^c_a \Delta_a G}. \tag{S22}$$

Gears for which $m^c_a = 0$ have a detrimental effect on the transduction efficiency since they contribute solely to the numerator with the term $j_c m^c_b \Delta_b G$, which is negative according to Eq. (S18). Thus, in the following, we restrict to the case where $m^c_a \neq 0$ for all gears and we define their forward direction as the one having $m^c_a > 0$. We can then rewrite Eq. (S22) as:

$$\eta = \frac{\sum_c j_c m^c_a \eta_c}{\sum_c j_c m^c_a}. \tag{S23}$$

Given that the Gibbs free energy change of each conformal gear is $\Delta_c G = m^c_a \Delta_a G + m^c_b \Delta_b G$ and that $-j_c \Delta_c G > 0$ from Eq. (S18), the relation between η_c and j_c is:

$$\begin{aligned} \eta_c < 1 &\iff \Delta_c G < 0 \iff j_c > 0, \\ \eta_c > 1 &\iff \Delta_c G > 0 \iff j_c < 0. \end{aligned} \tag{S24}$$

In words, whether η_c is thermodynamically feasible or not determines the sign of j_c in a conformal decomposition.

Starting from Eq. (S23), we divide the set of conformal gears into feasible and unfeasible, that is, $\{c\} = \{c'\} + \{c''\}$ with $\eta_{c'} < 1$ and $\eta_{c''} \geq 1$. From Eq. (S24), we have $j_{c'} > 0$ and $j_{c''} < 0$, which implies that the coefficients $r_{c'} = j_{c'} m^{c'}_a$ and $q_{c''} = -j_{c''} m^{c''}_a$ are both nonnegative since $m^c_a > 0$. We can express the efficiency in Eq. (S23) as:

$$\eta = \frac{\sum_{c'} r_{c'} \eta_{c'} - \sum_{c''} q_{c''} \eta_{c''}}{\sum_{c'} r_{c'} - \sum_{c''} q_{c''}}. \tag{S25}$$

We first show that every $q_{c''} \neq 0$ negatively affects the efficiency compared to the case where the same $q_{c''}$ is zero. To do this, we rewrite the efficiency as follows.

$$\eta = \frac{C - q_{c''} \eta_{c''}}{D - q_{c''}}, \tag{S26}$$

where all the other terms have been reabsorbed into the constants C and D. Both the numerator and the denominator are positive since the CRN is performing transduction. In addition, $C < D\,\eta_{c''}$ must hold to have $\eta < 1$, which is enough to prove that $\eta < C/D$. Repeating this argument for all the conformal gears in $\{c''\}$, one obtains:

$$\eta \leq \frac{\sum_{c'} r_{c'}\eta_{c'}}{\sum_{c'} r_{c'}}. \tag{S27}$$

The RHS is simply a weighted average with positive coefficients and therefore it is

$$\eta \leq \max\, \eta_{c'}, \tag{S28}$$

from which Eq. (S19) follows.

VII. SELF-REGULATING CRN

We report here the forward and backward reaction fluxes assigned to the model in Fig. 2 in the main text:

$$r_1 : E_1 + S \underset{k_r[E_1^*][\bar{S}]}{\overset{k_r[E_1][S]}{\rightleftharpoons}} E_1^* \qquad r_2 : E_2^* + S \underset{k_r[E_2][\bar{S}]}{\overset{k_r[E_2^*][S]}{\rightleftharpoons}} E_2,$$
$$e_1 :\; E_1 + S \underset{k_e[E_1][X]}{\overset{k_e[E_1][S]}{\rightleftharpoons}} X + E_1,$$
$$e_2 :\; E_2 + ADP + P_i + S \underset{k_e\exp(\Delta_{ATP}G)[E_2][X]}{\overset{k_e[E_2][S]}{\rightleftharpoons}} X + ATP + E_2,$$
$$d :\; ADP + P_i + X \underset{k_d\exp(\Delta_{ATP}G)[P]}{\overset{k_d\exp(\Delta_{SP}G^0)[X]}{\rightleftharpoons}} P{+}ATP. \tag{S29}$$

From the above rates and the total concentration of enzymes $L = [E_i^*] + [E_i]$, it follows

$$[E_1] = \frac{[\bar{S}]}{[\bar{S}] + [S]}L \quad \text{and} \quad [E_2] = \frac{[S]}{[\bar{S}] + [S]}L. \tag{S30}$$

We assume $k_d \gg k_e L$ so that reaction d is much faster than e_1 and e_2. In this limit, we can effectively consider it to be at equilibrium:

$$[X] \approx e^{\Delta_{ATP}G - \Delta_{SP}G^0}[P]. \tag{S31}$$

From the knowledge of $[X]$, one can derive the reaction currents J_{e_1} and J_{e_2}.

$$\begin{aligned} J_{e_1} &= k_e[E_1]([S] - [X]) = K\frac{[\bar{S}]}{[S] + [\bar{S}]}(e^{\Delta_{SP}G} - e^{\Delta_{ATP}G}),\\ J_{e_2} &= k_e[E_2]([S] - e^{\Delta_{ATP}G}[X]) = K\frac{[S]}{[S] + [\bar{S}]}(e^{\Delta_{SP}G} - e^{2\Delta_{ATP}G}),\end{aligned} \tag{S32}$$

where $K = k_e[L][P]e^{-\Delta_{SP}G^0}$. The transduction efficiency is then:

$$\eta = \frac{J_{e_1} + 2J_{e_2}}{J_{e_1} + J_{e_2}}\frac{\Delta_{ATP}G}{\Delta_{SP}G} = \frac{[\bar{S}](e^{\Delta_{SP}G} - e^{\Delta_{ATP}G}) + 2[S](e^{\Delta_{SP}G} - e^{2\Delta_{ATP}G})}{[\bar{S}](e^{\Delta_{SP}G} - e^{\Delta_{ATP}G}) + [S](e^{\Delta_{SP}G} - e^{2\Delta_{ATP}G})}\frac{\Delta_{ATP}G}{\Delta_{SP}G}, \tag{S33}$$

that can be rewritten as:

$$\eta = \frac{e^{\Delta_{SP}\bar{G}}(e^{\Delta_{SP}G} - e^{\Delta_{ATP}G}) + 2e^{\Delta_{SP}G}(e^{\Delta_{SP}G} - e^{2\Delta_{ATP}G})}{e^{\Delta_{SP}\bar{G}}(e^{\Delta_{SP}G} - e^{\Delta_{ATP}G}) + e^{\Delta_{SP}G}(e^{\Delta_{SP}G} - e^{2\Delta_{ATP}G})}\frac{\Delta_{ATP}G}{\Delta_{SP}G}, \tag{S34}$$

where $\Delta_{SP}\bar{G} = \mu_S^0 + \log[\bar{S}] - \mu_P$. In Fig. 3a, the parameter q is the value at which $[E_1] = [E_2]$ and $[S] = [\bar{S}]$. Thus:

$$q = \frac{\Delta_{ATP}G}{\Delta_{SP}\bar{G}}. \tag{S35}$$

Finally, the output power is given by:

$$P = (J_{e_1} + 2J_{e_2})\,\Delta_{ATP}G = K\left(\frac{[\bar{S}]}{[S]+[\bar{S}]}(e^{\Delta_{SP}G} - e^{\Delta_{ATP}G}) + 2\frac{[S]}{[S]+[\bar{S}]}(e^{\Delta_{SP}G} - e^{2\Delta_{ATP}G})\right)\Delta_{ATP}G, \tag{S36}$$

which can be rewritten as

$$P = K\left(\frac{e^{\Delta_{SP}\bar{G}}}{e^{\Delta_{SP}\bar{G}} + e^{\Delta_{SP}G}}(e^{\Delta_{SP}G} - e^{\Delta_{ATP}G}) + 2\frac{e^{\Delta_{SP}G}}{e^{\Delta_{SP}\bar{G}} + e^{\Delta_{SP}G}}(e^{\Delta_{SP}G} - e^{2\Delta_{ATP}G})\right)\Delta_{ATP}G. \tag{S37}$$

P_{max} mentioned in the text is given by:

$$P_{\mathrm{max}} = \begin{cases} K\Delta_{ATP}G\left[(e^{\Delta_{SP}G} - e^{\Delta_{ATP}G}) + 2(e^{\Delta_{SP}G} - e^{2\Delta_{ATP}G})\right] & \text{if } \Delta_{ATP}G/\Delta_{SP}G < \frac{1}{2}, \\ K\Delta_{ATP}G\,(e^{\Delta_{SP}G} - e^{\Delta_{ATP}G}) & \text{if } \Delta_{ATP}G/\Delta_{SP}G \geq \frac{1}{2} \end{cases}. \tag{S38}$$

This is the maximum power that can be achieved with the constraint $[E_1]$, $[E_2] \leq L$. It corresponds to using all the enzymes available before $\Delta_{ATP}G/\Delta_{SP}G < 1/2$ and, after, only $[E_1] = L$ since gear $\boldsymbol{\psi}_2$ works in the reverse direction, $J_{e_2} < 0$, and thus is counterproductive. In the main text plots, we set $K = 1$.

---

[1] M. Polettini and M. Esposito, The Journal of Chemical Physics **141**, 024117 (2014), ISSN 0021-9606.
[2] M. Terzer and J. Stelling, Bioinformatics **24**, 2229 (2008), ISSN 1367-4803.
[3] J. Zanghellini, D. E. Ruckerbauer, M. Hanscho, and C. Jungreuthmayer, Biotechnology Journal **8**, 1009 (2013).
[4] S. Müller and G. Regensburger, Frontiers in Genetics **7** (2016), ISSN 1664-8021.
[5] T. L. Hill, in *Free Energy Transduction in Biology*, edited by T. L. Hill (Academic Press, 1977), ISBN 978-0-12-348250-1.
